# Pilot study of long‐term anaesthesia in broiler chickens

**DOI:** 10.1111/vaa.12305

**Published:** 2015-10-09

**Authors:** Peter M O'Kane, Ian F Connerton, Kate L White

**Affiliations:** ^1^ Division of Food SciencesSchool of BiosciencesUniversity of NottinghamSutton BoningtonUK; ^2^ School of Veterinary Medicine and ScienceUniversity of NottinghamSutton BoningtonUK

**Keywords:** anaesthesia, avian, chicken, ligated loop, monitoring

## Abstract

**Objective:**

To provide stable anaesthesia of long duration in broiler chickens in order to perform a terminal caecal ligated loop procedure.

**Study design:**

Prospective experimental study.

**Animals:**

Seven clinically healthy broiler chickens (*Gallus domesticus*) aged 27–36 days, weighing 884–2000 g.

**Methods:**

Anaesthesia was induced and maintained with isoflurane in oxygen. All birds underwent intermittent positive pressure ventilation for the duration. End‐tidal carbon dioxide_,_ peripheral haemoglobin oxygen saturation, heart rate and oesophageal temperature were monitored continuously. All birds received intraosseous fluids. Butorphanol (2 mg kg^−1^) was administered intramuscularly at two hourly intervals. Euthanasia by parenteral pentobarbitone was performed at the end of procedure.

**Results:**

Stable anaesthesia was maintained in four chickens for durations ranging from 435 to 510 minutes. One bird died and one was euthanized after 130 and 330 minutes, respectively, owing to surgical complications and another died from anaesthetic complication after 285 minutes.

**Conclusions and clinical relevance:**

Long‐term, stable anaesthesia is possible in clinically healthy chickens, provided complications such as hypothermia and hypoventilation are addressed and vital signs are carefully monitored. There are no known previous reports describing monitored, controlled anaesthesia of this duration in chickens.

## Introduction

Much is written in clinical texts and the literature about recommendations for safe anaesthesia in avian patients, but little has been published concerning long‐term anaesthesia. Fedde (1978) reviewed many of the agents used in avian anaesthesia, including phenobarbitone, which provided anaesthesia of 24 hours’ duration. However, few details are given regarding the stability or monitoring of anaesthesia in these reports.

We report the results of a pilot study in which long‐term anaesthesia was required for a caecal ligated loop experiment studying bacteriophage therapy for *Campylobacter jejuni* (Connerton et al. 2011). The procedure was based on that reported by Van Deun et al. (2008), but with the objective of longer‐term anaesthesia followed by euthanasia rather than recovery.

## Materials and methods

### Animals

A total of seven 1‐day‐old male broiler (Ross 308) chickens were obtained from a commercial hatchery and reared in a biosecure environment until the day of procedure, when the birds were between 27 and 36 days of age, in accordance with the Home Office code of practice for the housing and care of animals used in scientific procedures. Birds were group housed until 20 days of age and were individually caged thereafter. Feed and water were available *ad libitum* with a 12 hour light/dark cycle. All birds were considered to be healthy at the preanaesthesia clinical examination.

This study was carried out in accordance with UK and EU legislation. All procedures were approved by the Local Ethics Committee of the University of Nottingham and performed under Home Office licence.

### Anaesthetic protocol

Feed was withdrawn on the morning of the procedure, the birds were weighed and their crops palpated to confirm no ingesta were present. While restraining birds manually with a towel, anaesthesia was induced via a mask attached to an Ayre's T‐piece circuit through which isoflurane (5%; IsoFlo; Abbott, UK) was delivered from an agent‐specific vaporizer. Once anaesthetized, each bird was intubated with an uncuffed orotracheal tube (Portex; Smiths Medical, UK) with internal diameter 2.5–4 mm, depending on bird size. Anaesthesia was maintained with isoflurane in oxygen and intermittent positive pressure ventilation was performed using a pressure‐limited ventilator (SAV03; Vetronic Services, UK). The trigger point was set to achieve normal inspiratory depth and the expiratory time was adjusted to maintain an end‐tidal carbon dioxide (Pe′CO_2_) target of 35–45 mmHg (4.7–6.0 kPa). Depth of anaesthesia was assessed using cardiovascular parameters and reflexes and isoflurane vaporizer settings were varied between 2.5% and 3%. Each bird was positioned in dorsal recumbency between warm water‐filled gloves on an electronic heat mat and foam wedge at a head‐up angle of approximately 10°. Heart rate (HR) and peripheral haemoglobin oxygen saturation (SpO_2_) were measured with a VM 2500 veterinary CO_2_/SpO_2_ monitor (Viamed, UK) with the pulse oximeter probe placed on a wing web or between toes. The Pe′CO_2_ and ventilation rate (*f*_R_) were measured on the same unit. A flexible thermometer probe attached to a monitoring console (Minimon 7138B; Kontron, UK) was introduced oesophageally to approximately the level of the heart. Body temperature, SpO_2_, Pe′CO_2_, HR and *f*_R_ were monitored continuously and recorded every 15 minutes.

A 21 g hypodermic needle was inserted into the medullary cavity of the tibiotarsus to deliver lactated ringers (Vetivex 11; Dechra, UK) solution at 10 mL kg^−1^ hour^−1^.

Butorphanol (Torbugesic; Pfizer, UK) was administered intramuscularly, into the superficial pectoral or thigh, at a single dosage of 1 mg kg^−1^ in the first bird and 2 mg kg^−1^ every 2 hours in the subsequent six birds.

### Surgical procedure

A midline coeliotomy was performed with parasternal flap extension to allow exteriorization of the intestines and caeca for ligation, sampling and injection. Further sampling was performed every 1–2 hours for a total of 6 hours. Between samplings the viscera were returned to the body cavity and the body wall was temporarily apposed.

All birds were euthanized at the end of the procedure with an overdose of parenteral pentobarbitone.

## Results

Ages, weights and monitored parameters are shown in Table 1. Birds 2, 3, 5 and 7 survived for the duration required for the experiment. Bird 1 died following a surgical complication during the coeliotomy. In response to the application of towel clamps to temporarily appose the body wall after returning the viscera to the abdominal cavity, the bird became tachycardic and the isoflurane concentration was increased, but the bird died very soon thereafter. The technique was refined by using stay sutures to achieve apposition and avoiding direct coelomic irrigation with lavage fluid in subsequent surgeries. Bird 4 was euthanized after 330 minutes following detection of caecal thromboemboli. We believe this occurred following a potential torsion event after viscera were returned to the body cavity. Care was taken to avoid this in subsequent procedures.

**Table 1:** Monitored variables in broiler chickens undergoing anaesthesia (median ± standard deviation)

Bird	Age (days)	Weight (g)	Anaesthetic duration (minutes)	SpO_2_ (%)	HR (beats minute^−1^)	Ventilation rate (breaths minute^−1^)	Pe′CO_2_ [mmHg (kPa)]	Oesophageal temperature (°C)
1	27	884	130	99 ± 1	251 ± 70	29 ± 7	44 ± 12 (6 ± 2)	38.8 ± 1.0
2	29	1100	480	99 ± 1	252 ± 38	16 ± 3	37 ± 13 (5 ± 2)	39.6 ± 0.8
3	30	1160	435	99 ± 0	284 ± 22	20 ± 4	47 ± 10 (6 ± 1)	39.6 ± 0.9
4	31	1140	330	99 ± 0	305 ± 30	19 ± 6	44 ± 11 (6 ± 1)	40 ± 1.0
5	34	1300	450	96.5 ± 4	294 ± 26	18 ± 5	45 ± 8 (6 ± 1)	40.7 ± 0.8
6	35	2000	285	99 ± 0	241 ± 44	19 ± 31	47 ± 13 (6 ± 2)	39.2 ± 1.2
7	36	1585	510	99 ± 0	272 ± 45	20 ± 5	38 ± 10 (5 ± 2)	40.6 ± 0.9

Birds 2, 3, 5 and 7 were euthanized on completion of the experiment. Birds 1 and 6 died during anaesthesia and Bird 4 was euthanized early as a result of a surgical complication

SpO_2_, peripheral haemoglobin oxygen saturation; HR, heart rate; pe′CO_2_, end‐tidal carbon dioxide.

Bird 6 died unexpectedly after 285 minutes of anaesthesia; this was noted as a sudden drop in Pe′CO_2_ followed by loss of pulse oximeter trace and palpable heartbeat. No change in monitoring parameters were noted prior to this occurrence.

Despite reducing airway pressure on entry of the body cavity and attempting to pack off with moistened swabs, at least one abdominal air sac was ruptured in all of the birds. This occurred either on initial approach to the body cavity, where air sacs easily ballooned and ruptured if pressure was not reduced, or after viscera were manipulated.

Airway pressure did not exceed 15 cmH_2_O in any of the birds; once the body cavity was opened the pressure was reduced to as low as 4 cmH_2_O to minimize volutrauma to the air sacs. The ventilation frequency rate was adjusted to maintain adequate ventilation as determined by Pe′CO_2_.

The endotracheal tube was replaced at least once per procedure as routine and it was changed immediately if any obstruction was suspected. Thick mucus was often present after 2–3 hours of anaesthesia.

## Discussion

A ligated loop study was selected for this experiment as it removes many variables encountered in alternative experimental designs. These include variation in initial *Campylobacter* colonization levels, gastrointestinal transit times and influences of microbiota encountered en route to the caeca. This approach should greatly reduce the number of animals required to obtain significant results as it removes inter‐animal variation. This is in keeping with the principle of replacement, refinement and reduction of animals in research (Russell & Burch 1959). The authors are unaware of any published reports describing a monitored, controlled anaesthesia of this duration in chickens. Several older texts describe ligated loop studies, but the anaesthesia is often not described in detail. We report these results to demonstrate that long‐term anaesthesia for this caecal ligation model is viable, so that others may use it in future.

No blood haematological or biochemical testing was performed, as these were all young, clinically healthy birds and results would have been unlikely to change the protocol.

Once anaesthesia was induced, intermittent positive pressure ventilation was initiated with no resistance or bucking of the ventilator. There was therefore no requirement for neuromuscular blockade. Butorphanol was included in the protocol as it has been demonstrated to have an isoflurane‐sparing effect in Psittaciformes (Curro et al. 1994). The pharmacokinetics of butorphanol have recently been described in broilers by Singh et al. (2011), informing our selected dose of 2 mg kg^−1^ every 2 hours.

Pulse oximetry for the estimation of haemoglobin oxygen saturation has been widely considered as unreliable in avian species (Edling 2006). The equipment is calibrated for mammalian, not avian, haemoglobin and tissues and tends to underestimate the haemoglobin saturation in birds (Schmitt et al. 1998). The Viamed pulse oximeter used in this study provided a very consistent trace and gave an audible alarm when the trace was lost. Validating SpO_2_ data was not possible in this pilot study, but this could be performed in future studies. The pulse oximeter probe from the older Kontron monitor (Minimon 7138B) provided no trace or SpO_2_ reading.

Bird 6, which died after 285 minutes of anaesthesia, was the heaviest and most muscled of the chickens anaesthetized. The ‘sudden death’ syndrome (SDS) of broiler chickens tends to affect faster‐growing birds and although the aetiology is poorly defined it may be associated with cardiac arrhythmias (Crespo & Shivaprasad 2013). Given that this bird may have had the lowest cardiorespiratory reserve capacity within our cohort, we believe SDS is a possibility. No gross lesions were apparent at post‐mortem, which can be consistent with SDS.

Clinical texts frequently emphasize the requirement for speed, as avian patients requiring anaesthesia for procedures are rarely healthy (Edling 2006). Long‐term, stable anaesthesia does appear to be possible in healthy chickens. As these were terminally anaesthetized for ethical reasons, we have no data on recovery and survival after the procedures. The anticipated problems of hypothermia, hypoventilation and regurgitation were avoided or managed, and monitored parameters were within acceptable physiological limits. Studies such as that by Fedde et al. (1998) have demonstrated the HR in conscious broiler chickens to be in the region of 360 beats minute^−1^.

The duration of preoperative fasting in avian patients is controversial, with the aim being to balance the avoidance of regurgitation during induction with the maintenance of adequate energy reserves for a long procedure (Edling 2006). A small degree of regurgitation was noted mid‐procedure in Bird 6, which had the shortest feed withdrawal time of 2 hours; however, the crop was palpably empty even when the feed was removed. The material was removed with cotton swabs, and was considered unrelated to the anaesthetic death as no material was noted in the trachea at post‐mortem examination.

As described elsewhere in the literature (Edling 2006), air sacs were ruptured during this procedure. Isoflurane pollution of the environment is inevitable, and therefore adequate ventilation of the operative area is essential and charcoal‐filtered surgical masks should be considered.

Based on the results of this pilot study, a larger‐scale experiment can be designed with further refinements, including arterial blood gas analysis to validate the SpO_2_ and Pe′CO_2_ monitoring equipment. This will expand the data set of normal values for broiler chickens undergoing anaesthesia and supplement the findings of others that Pe′CO_2_ is accurately correlated with arterial concentrations (Edling 2006).

## References

[vaa12305-bib-0001] Connerton PL , Timms AR , Connerton IF (2011) Campylobacter bacteriophages and bacteriophage therapy. J Appl Microbiol 111, 255–265. 21447013 10.1111/j.1365-2672.2011.05012.x

[vaa12305-bib-0002] Crespo R , Shivaprasad HL (2013) Developmental, metabolic, and other noninfectious disorders In: Diseases of Poultry (13th edn). Swayne DE, Glisson JR, McDougald LR, Nolan LK, Suarez DL, Nair VL (eds). Wiley‐Blackwell, USA pp. 1233–1270.

[vaa12305-bib-0003] Curro TG , Brunson DB , Paul‐Murphy J (1994) Determination of the ED50 of Isoflurane and Evaluation of the Isoflurane‐Sparing Effects of Butorphanol and Flunixin Meglumine in Psittaciformes. Proceedings of the Association of Avian Veterinarians, Reno, NV, USA pp. 17–19. 10.1111/j.1532-950x.1994.tb00502.x 7839598

[vaa12305-bib-0004] Edling TM (2006) Updates in anesthesia and monitoring In: Clinical Avian Medicine. Harrison GJ, Lightfoot TL (eds). Spix, USA pp. 747–760.

[vaa12305-bib-0005] Fedde MR (1978) Drugs used for avian anesthesia: a review. Poult Sci 57, 1376–1399. 364455 10.3382/ps.0571376

[vaa12305-bib-0006] Fedde MR , Weigle GE , Wideman RF (1998) Influence of feed deprivation on ventilation and gas exchange in broilers: relationship to pulmonary hypertension syndrome. Poult Sci 77, 1704–1710. 9835347 10.1093/ps/77.11.1704

[vaa12305-bib-0007] Russell WMS , Burch RL (1959) The Principles of Humane Experimental Technique. Methuen, London, UK.

[vaa12305-bib-0008] Schmitt PM , Göbel T , Trautvetter E (1998) Evaluation of pulse oximetry as a monitoring method in avian anesthesia. J Avian Med Surg 12, 91–99.

[vaa12305-bib-0009] Singh PM , Johnson C , Gartrell B , et al. (2011) Pharmacokinetics of butorphanol in broiler chickens. Vet Rec 168, 588. 21628341 10.1136/vr.d1191

[vaa12305-bib-0010] Van Deun K , Pasmans F , Ducatelle R , et al. (2008) Colonization strategy of *Campylobacter jejuni* results in persistent infection of the chicken gut. Vet Microbiol 130, 285–297. 18187272 10.1016/j.vetmic.2007.11.027

